# Therapeutic Potential of Menstrual Blood-Derived Stem Cell Transplantation for Intrauterine Adhesions

**DOI:** 10.3389/fsurg.2022.847213

**Published:** 2022-02-22

**Authors:** Yantao He, Yanhua Han, Yun Ye

**Affiliations:** ^1^Department of Gynecology and Obstetrics, Zhongshan City People's Hospital, Zhongshan, China; ^2^Centre for Reproductive Medicine, Zhongshan City People's Hospital, Zhongshan, China

**Keywords:** intrauterine adhesion, Asherman syndrome, menstrual blood-derived stem cells, transplantation, angiogenesis

## Abstract

An increasing number of women experience intrauterine adhesion as a result of intrauterine operations, such as induced abortion, which can cause infertility, recurrent abortion and amenorrhea. Although some strategies have been applied clinically, such as hysteroscopy adhesiolysis of intrauterine adhesions, the results have not been promising. As regenerative medicine develops, research on menstrual blood-derived stem cell transplantation is increasing due to the properties of these cells, including self-renewal, differentiation, angiogenesis, anti-inflammation and immunomodulation. As a result, menstrual blood-derived stem cells may be an ideal cell source for the treatment of intrauterine adhesion. Excitingly, it has been reported that autologous menstrual blood stem cells could recovery injured endometrium and improve infertility in patients with refractory intrauterine adhesion. In this review, we discuss the possible potential of menstrual blood-derived stem cell transplantation for intrauterine adhesion, including the antifibrosis, angiogenesis, anti-inflammation and immunoregulation properties of the cells, which brings hopes for clinical therapy.

## Background

Intrauterine adhesion (IUA), also named as Asherman syndrome, refers to endometrial fibrosis caused by the damage of endometrium basal layer caused by repeated improper intrauterine operation and infection, which leads to partial or complete adhesion or even occlusion of uterus. IUA is characterized by symptoms such as recurrent miscarriage, fertile disorder, hypomenorrhea, amenorrhea and pregnancy complications (1). The pathogenesis of IUA is as follows: when the endometrium is repeatedly damaged, the basal layer of the endometrium cannot regenerate and is replaced by a large number of monolayer epithelium and fibrous tissue. Endometrium atrophy, rarefied, gland inactive, low response to hormone stimulation, blurred boundary between functional layer and basal layer, resulting in adhesion formation. IUA is mostly associated with trauma, infection, and genetic susceptibility, and contributes to secondary infertility in women (2). A main cause of IUA is curettage, such as post-abortion or miscarriage curettage, which may injure the endometrium. Additionally, tuberculosis and some infections are strong risk factors for IUA since the invasion of viruses and other microorganisms could induce fibrosis and inhibit the repair of the endometrium. Presently, hysteroscopy adhesiolysis combined with hormonal therapy is the main treatment for removing the visible IUA (3). At present, the traditional treatment has a good effect on mild and moderate IUA, but for patients with severe IUA, pregnancy complications often occur after hysteroscopic adhesiolysis, such as recurrent miscarriage, earlier delivery and abnormal placental development on account of injured endometrium (4). Several researchers have paid much attention to a new adult stem cell, which is able to regenerate endometrial tissue, although more studies are needed (5). Therefore, the application of stem cells from the endometrium, such as menstrual blood-derived stem cell (MenSCs), is a research hotspot for endometrial regeneration. At present, MenSCs have been used in the treatment of polycystic ovary syndrome, myocardial infarction, type I diabetes and other diseases.

The decrease or absence of the number of endometrial stem cells and local inflammatory environment are the fundamental reasons for the re-adhesion after IUA and separation. Given their pluripotency and low immunogenicity, MenSCs are believed to have therapeutic potential for IUA. Study showed that MenSCs could differentiate into endometrial cells when managed *in vitro* and reproduct endometrial tissue in mice with IUA *in vivo* (5, 6). Another study revealed that MenSCs transplantation combined with estrogen could improve endometrial abnormalities (7). MenSCs have a positive effect on antifibrosis, angiogenesis, and anti-inflammation in IUA. Moreover, another study revealed that reproductive endometrial tissue and vessel were found but fibrosis was decreasing in the uterus of IUA because of stem cell transplantation and exosome treatments, and researchers found that exosomal treatment contributed to recovery injured endometrial tissue caused by IUA (8). These studies demonstrate that MenSCs is a good candidate for the treatment of IUA. Autologous MenSCs have been transplanted to patients with refractory IUA for clinical therapy. The results revealed that patients with refractory IUA had thicker endometrium and prolonged menstrual duration managed by MenSCs transplantation, and some of them achieved clinical pregnancy (9). In that study, five out of 12 patients got pregnant, and in other words, there was a pregnancy rate of 41.7% (9). Another study also showed women with IUA had thicker significantly endometrium after autologous MenSCs transplantation (10). We summarized the efficacy of autologous MenSCs transplantation in patients with IUA in Table 1 (9, 10). The researches on MenSCs transplantation for the models of IUA are not enough, and clinical trials also need more attention, which could bring hopes for patients with IUA.

**Table 1:** The therapeutic effects of autologous MenSCs on patients with IUA.

**Patients, n**	**Age, years**	**Transplantation method**	**Isolated volume, ml**	**Transplantation numbers, million**	**Duration of menstruation before transplantation, day**	**Duration of menstruation after transplantation, day**	**Endometrial thickness before transplantation, mm**	**Endometrial thickness after transplantation, mm**	**Pregnancy rate, n (%)**
12	22–40	*in situ*	1	10	2.4 ± 0.7	5.3 ± 0.6	3.9 ± 0.9	7.5 ± 0.6	5 (41.7)
7	20–40	*in situ*	0.5	1	Unclear	Unclear	3.9 ± 1.03	6.7 ± 0.8	3 (43)

## Characteristics of MenSCs

**Figure 1:**
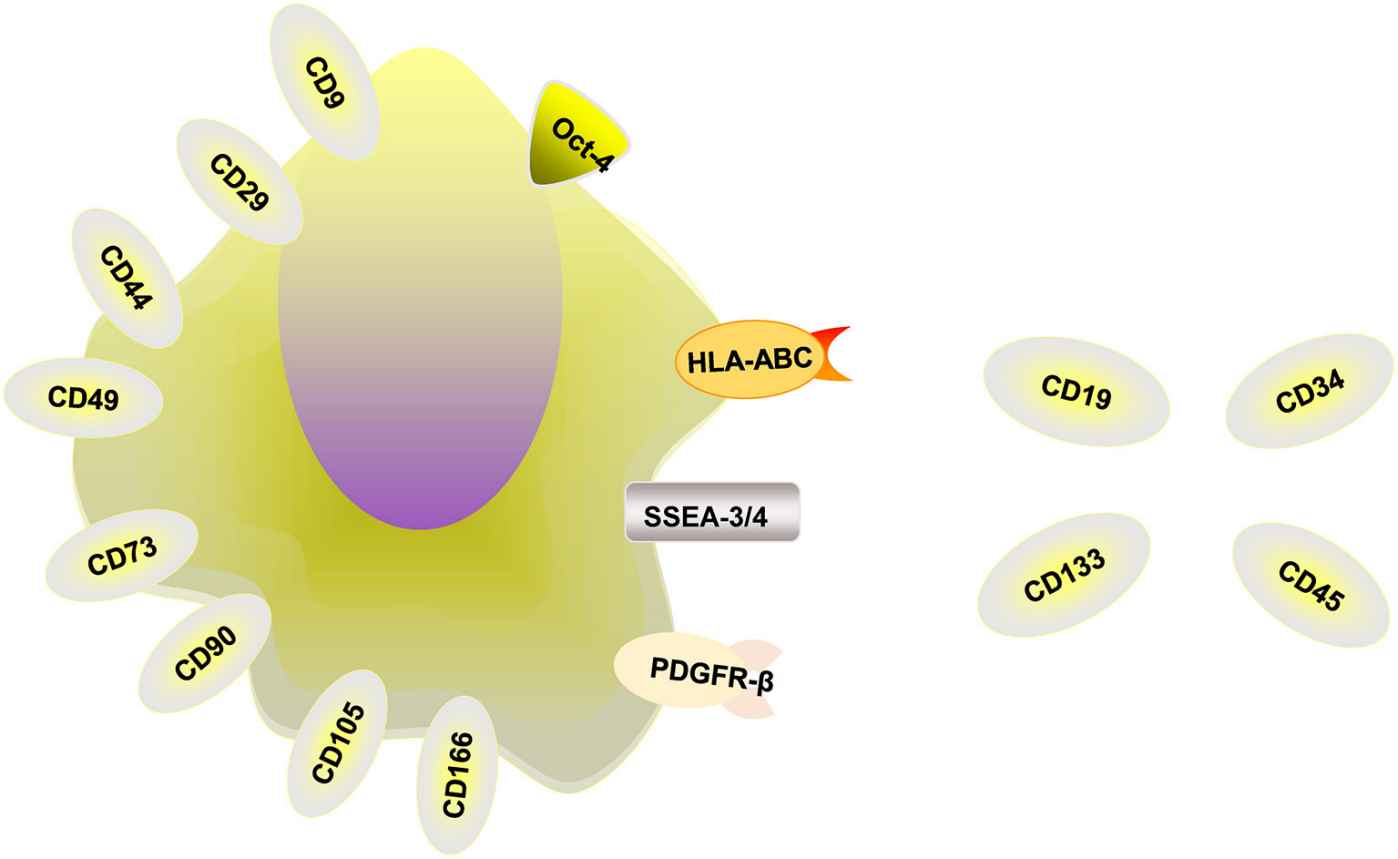
The relevant markers of MenSCs. MenSCs express CD9, CD29, CD44, CD49, CD73, CD90, CD105, and CD166, HLA-ABC, Oct-4, and SSEA-3/4 and PDGFR-β and unexpress CD19, CD34, CD45, CD133, and HLA-DR.

MenSCs obtained from the menstrual blood of women have attracted the attention of numerous researchers because of their many advantages (11, 12). MenSCs have beneficial properties, including ease of acquisition, non-invasive collection procedures, widespread expansion capacities, rapid amplification abilities, genomic stability and high proliferation rates without being tumorigenic or immunogenic (13). Some researchers have found that MenSCs are multipotent, with the potential to differentiate into germ cells, endometrial cells, and endothelial, osteogenic, adipocytic, cardiomyocytic, respiratory epithelial, neurocytic, cartilaginous, myocytic, hepatic, and pancreatic cell lines (14, 15). In addition, these cells can secrete cytokines to induce antifibrosis, angiogenesis, anti-inflammation and immunoregulation. MenSCs are thought to be isolated from menstrual blood instead of the endometrium, express some cell-surface markers such as CD166, CD105, CD90, CD73, CD49, CD44, CD29, CD9, and HLA-ABC and differentiate into chondrocytes, osteocytes, and adipocytes (16). Studies have shown that the positive expression rates of MenSCs in the third generation were CD29 [(99.13 ± 0.19) %], CD44 [(98.97 ± 0.34) %], CD73 [(99.8 ± 0.08) %] and CD105 [(99.17 ± 0.34)]%, and the positive expression rates were all above 95%, and the expression rate of CD90 was (72.43 ± 0.76) % (17). MenSCs can also express the embryonic stem cell markers Oct-4 and SSEA-3/4 and PDGFR-β (6, 18). Whether MenSCs express embryonic markers c-Kit (CD117) and SSEA-4 is under argument, and more studies are needed to explore whether these markers are expressed by MenSCs (16). MenSCs don't express HLA-DR, CD133, CD45, CD 34, and CD19 (19). The relevant markers are summarized in Figure 1. In addition, in order to evaluate the influence of menstrual blood storage time before MenSCs isolation on their vitality, the collected menstrual blood samples were divided into four equal parts and stored at 4°C. MenSCs were then isolated at 6, 24, 48, and 72 h, respectively. The results showed that there were no significant differences between MenSCs isolated after these different storage periods. Therefore, menstrual blood samples can be stored at 4°C for at least 3 days before further processing (20).

## Differentiation of MenSCs

**Figure 2:**
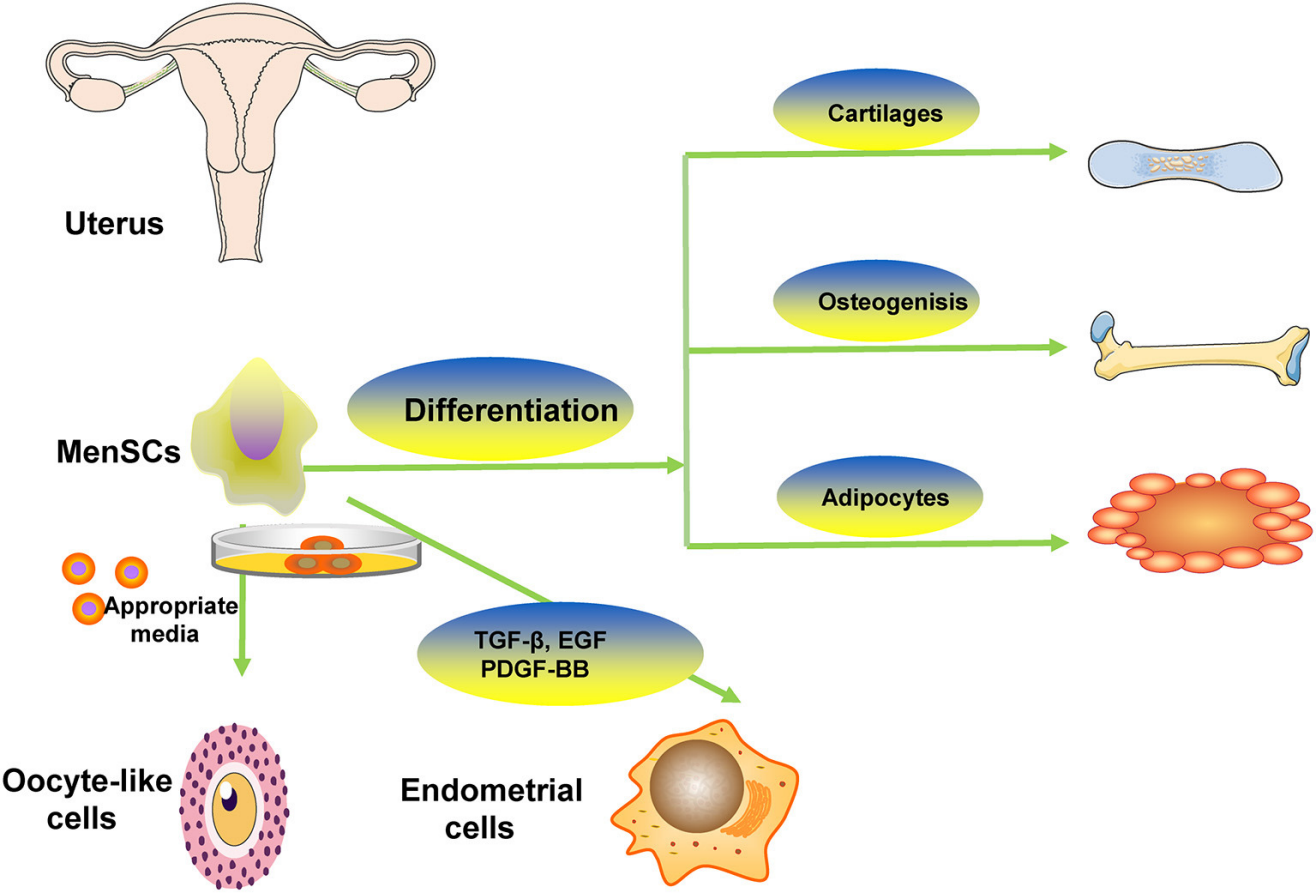
The representative differentiation of MenSCs. MenSCs differentiate into cartilaginous, adipocytic, osteogenic, oocyte-like cells.

Some studies showed that MenSCs had capacity of differentiating into different cells such as adipocytic, osteogenic, cardiogenic, cartilaginous, cardiomyocytic, muscle, neurogenic, glial-like, endothelial, oocyte-like, granulosa, respiratory epithelial, myocytic, hepatic, and pancreatic tissues (14, 15, 20). The representative differentiation of MenSCs is showed in Figure 2. Study revealed that MenSCs were able to differentiate into ovarian tissue-like cells when managed *in vitro* and that MenSCs treatment could repair injured ovary in animal models with premature ovarian failure (21). Another study showed that MenSCs could differentiate into oocyte-like cells and express follicle stimulating hormone receptor and luteinizing hormone receptor, which were oocyte-like cell markers, induced by appropriate media (22). Study revealed that MenSCs could differentiate into endometrial cells *in vitro via* appropriate medium contained with 17β-estradiol valerate, transforming growth factor-β (TGF-β)-1, epidermal growth factor (EGF), platelet-derived growth factor (PDGF)-BB (23). Some researchers found that there were some morphological changes in managed MenSCs, and they expressed oocyte-related genes (LHR, FSHR, STRA8, PRDM, STELLA, GDF9, SCP3, DDX4, and ZP2) in the 2^nd^ week of culture, suggesting the possibility of MenSCs differentiating into oocyte-like cells (24). To demonstrate the stem cell properties of MenSCs, studies have been conducted to test their pluripotency by culturing them with various types of differentiation media. The study found that MenSCs underwent adipogenic, osteogenic, cartilaginous, neural and cardiogenic differentiation, respectively, demonstrating their stem cell properties (20). The possible differentiation pathway is depicted in Figure 2.

## Antifibrotic Effects of MenSCs

**Figure 3:**
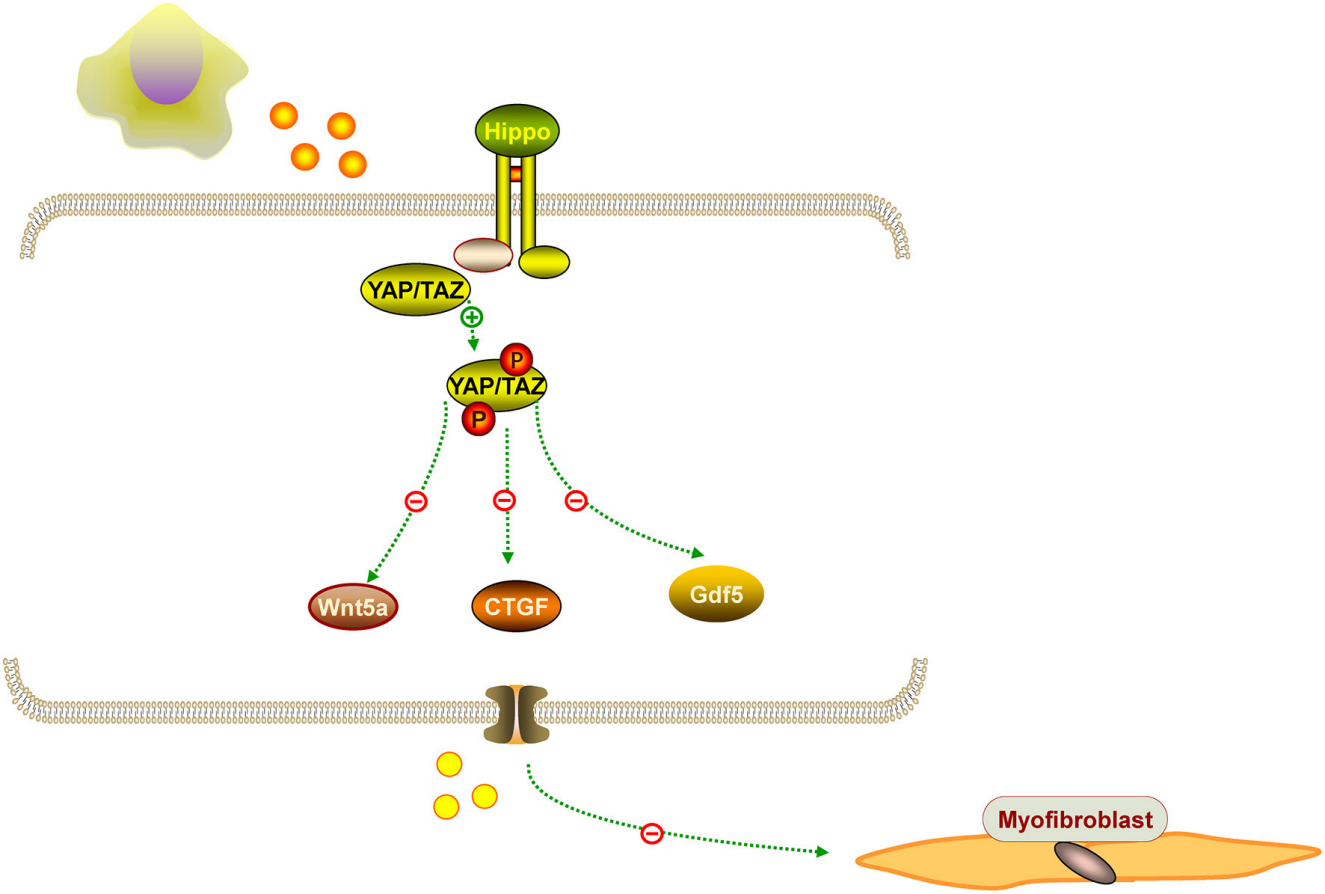
The representation of the antifibrotic effect of MenSCs. MenSCs could suppressed endometrial myofibroblast differentiation by Hippo/TAZ pathway stimulation, and the pathway can downstream factors CTGF, Wnt5a, and Gdf5.

IUA is characterized by an increased fibrotic area, thinner endometrium, fewer glands, and fewer microvessels. Hysteroscopy adhesiolysis cannot completely alleviate fibrosis. Surprisingly, MenSCs have antifibrotic effects in some diseases. For example, MenSCs can ameliorate liver fibrosis *via* paracrine mediators (25). Another study demonstrated that exosomal from MenSCs had a positive effect on pulmonary fibrosis by activating NLRP3 inflammasome and regulating mtDNA damage and ROS, which bring hopes for patients with fibrotic lung disease (26). Based on the fibrosis pathology, alleviating endometrial fibrosis is important for IUA treatment. It is known that myofibroblasts contribute to fibrosis formation. Another study showed that MenSCs inhibited endometrial myofibroblast differentiation by activation of the Hippo/TAZ pathway (27). Similarly, Fan et al. confirmed that MenSCs could markedly accelerate endometrial damage repair in an IUA rat model by Hippo signaling pathway stimulation, that the Hippo signaling pathway was the most significantly changed pathway, and that the expression of downstream factors CTGF, Wnt5a, and Gdf5 were significantly altered in the treatment groups (28). Researchers found that human amniotic epithelial cells upregulated MMP-8 expression to decrease collagen deposition in the uterine scar, and *in vitro* studies further confirmed an increase level of MMP-8 in hAECs cultured with hydrogen peroxide (28). We propose that MenSCs have antifibrotic effects on IUA, which needs further investigation. The representation of the antifibrotic effect of MenSCs is mentioned in Figure 3.

## Angiogenesis

**Figure 4:**
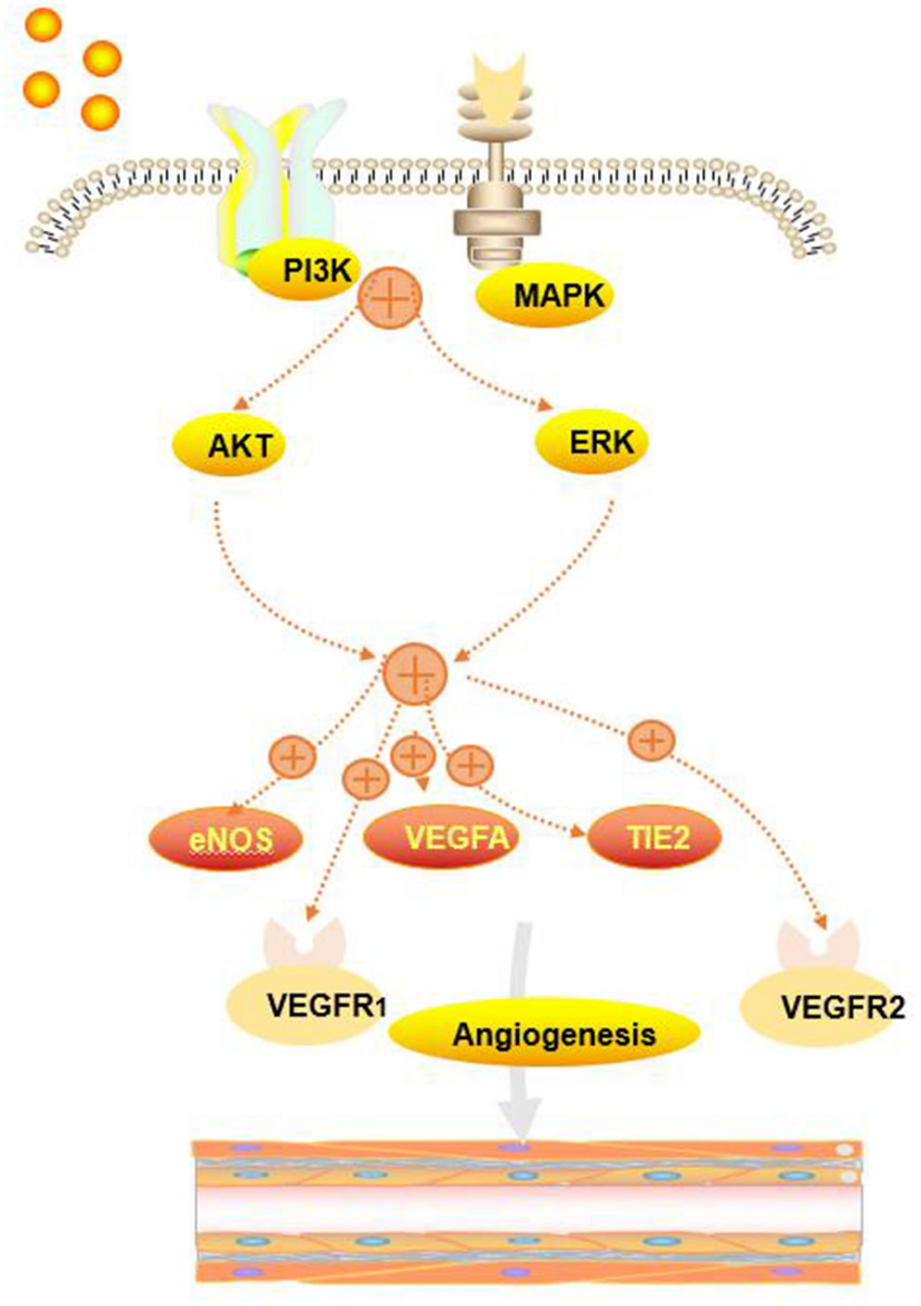
The angiogenesis of MenSCs. MenSCs could activate AKT and ERK pathways and induce the overexpression of eNOS, VEGFA, VEGFR1, VEGFR2, and TIE2.

Another function of MenSCs is their angiogenic effect. MenSCs show a strong angiogenic effect on endothelial cells both *in vitro* and *in vivo* (29). MenSCs were also able to induce angiogenesis *in vivo* (29). Zhang et al. found MenSCs-CM played an important role in angiogenesis in mice, which was associated with activation of AKT and ERK pathways, and overexpression of some factors such as VEGFR1, VEGFR2, eNOS, VEGFA, and TIE2 in HUVECs. These studies revealed MenSCs could revovery the injured endometrium and solve the fertile disorders in mice, which mainly depended on the angiogenesis induced by MenSCs (30). The angiogenesis of MenSCs in Figure 4.

## The Immunomodulatory and Anti-inflammatory Function of MenSCs

**Figure 5:**
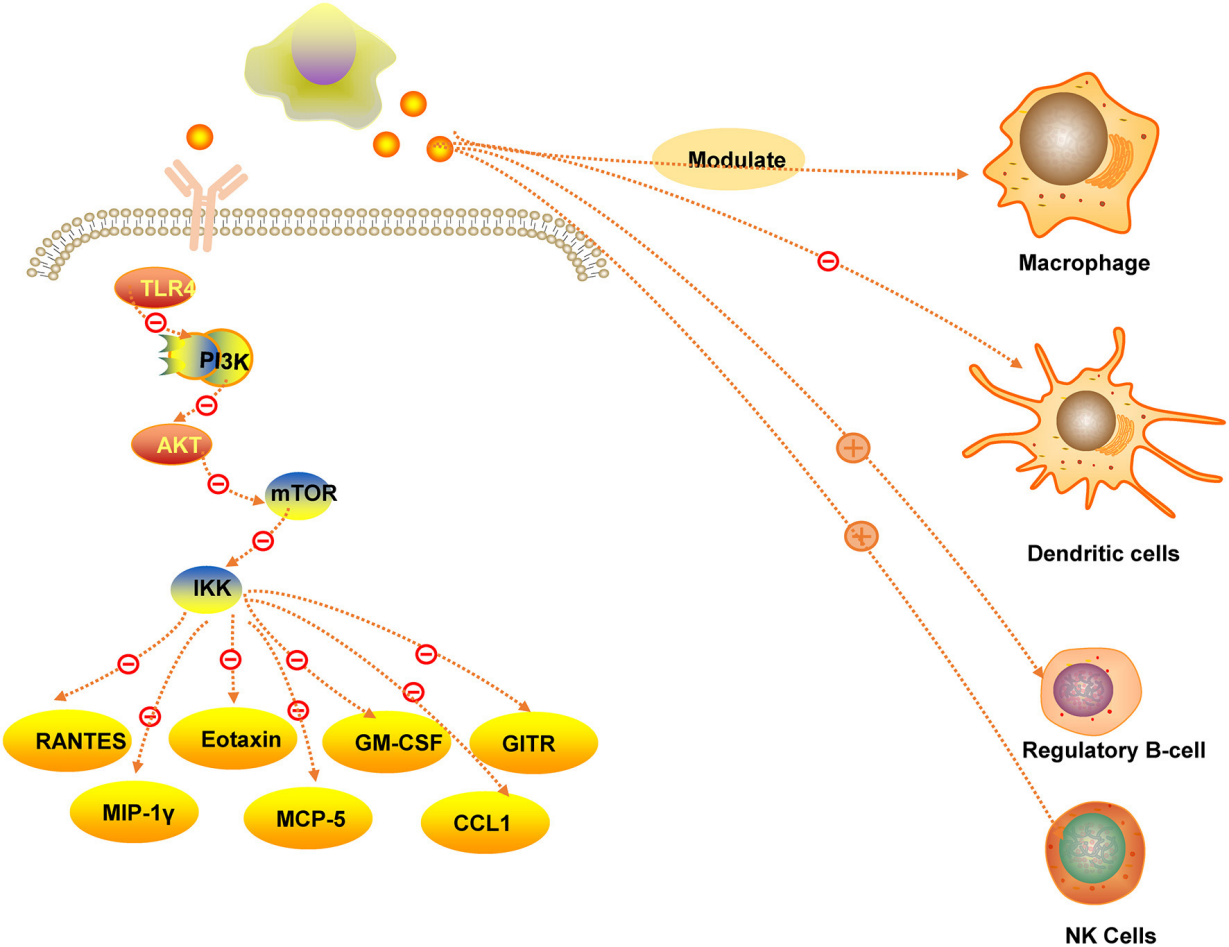
The immunomodulatory and anti-inflammatory function of MenSCs.

Menstrual blood stromal fibroblasts have the capacity of immunomodulatory and anti-inflammatory effects, which is similar with stem cells (6). MenSCs could suppress the apoptosis of MLE-12 cells *via* downregulating the expression of cytokines, such as GITR, GM-CSF, RANTES, MIP-1γ, eotaxin, MCP-5 and CCL1, which were involved with inflammatory reaction (31). In addition, *in vitro* studies demonstrated that MenSCs had a negative effect on macrophage bactericidal properties and the production of reactive oxygen intermediates, indicating that MenSCs effect the macrophage numbers, which provides a theoretical basis for clinical treatment in future (32). Moreover, MenSCs can have a negative effect on PI3K/Akt/mTOR/IKK signaling mediated by TLR4, leading to a decreasing of inflammatory cytokine, as p-NF-κBp65 could not be translocated into the nucleus (33). Another study showed that the maturation of human blood monocyte-derived dendritic cells was suppressed *via* interleukin−6 and interleukin−10(IL-10) secreted by MenSCs (34). The Immunomodulatory and anti-inflammatory function of MenSCs in Figure 5.

Studies have found that in a mouse model of ulcerative colitis, MenSCs reduced the infiltration of inflammatory cells, such as natural killer cells and macrophages, decreased the content of inflammatory factors such as tumor necrosis factor-α and IL-2, and increased the levels of inflammatory cytokines. IL-10 and IL-4 content (35). Researchers observed IL-10 and CXC chemokine receptor 4 (CXCR4) overexpress and numbers of regulatory B cells was increasing, suggesting that MenSCs have immunomodulatory properties (36). Some studies confirmed that nature killer cells (NK cells) were proliferating induced managed by MenSCs, but they were not when managed by IFN-γ/IL-1β-pretreated MenSCs (37). The researches on immunomodulation and anti-inflammation of MenSCs need more attention and efforts.

## Paracrine Effects of MenSCs

MenSCs secrete various chemokines that are crucial for the treatment of some diseases. One study confirmed that MenSCs had a positive effect on models of limb ischemia, by secreting some cytokines such MMP-3, MMP-10, IL-4 and hypoxia inducible factor-1 alpha (HIF-1α) (38). In addition, MenSCs secrete cytokines such as TGF-β2, EGF, PDGF and nitric oxide (NO) to enhance myocardial salvage and regeneration (39). Cuenca et al. confirmed that MenSCs could improve cutaneous regeneration by secreting cytokines, including MMP-3, MMP-10, PDGF, and angiopoietin (40). MenSCs express cytokines, including IL-8, IL-6, IGFBP-6, angiopoietin-2, ICAM-1, Axl and angiogenin to improve liver function and inhibit liver cell apoptosis (41). MenSCs also secrete TGF-β1 and rhTGF-β1 to play antitumor roles in cervical cancer, suggesting that MenSCs therapy is promising for the treatment of cervical cancer (42). MenSCs also secreted TSP-1, IGF-1 and stromal cell-derived factor-1(SDF-1) in a rat model of IUA. Another study showed that MenSCs have a positive effect on a rat model of IUA by secreting insulin-like growth factor (IGF)-1, thrombospondin-1 and SDF-1 (43).

## Safety of MenSCs Transplantation

MenSCs have properties of self-rewal and differentiation. Some researchers have conducted studies to determine whether MenSCs are likely to form tumors. One study demonstrated that MenSCs transplantation is safe for endometrial treatment for IUA (44). Some researchers believe that MenSCs have no risk of tumor formation and that MenSCs could have potential therapeutic effects on diseases through paracrine effects and immunomodulation (20). MenSCs has the advantages of convenient collection, non-invasiveness, no pain, multiple collection and no ethical disputes. Compared with bone marrow mesenchymal stem cells, MenSCs has higher proliferation ability, which is conducive to obtaining sufficient number of cells clinically in a short time. Some researchers evaluated the biosafety of MenSCs transplantation in animal model of IUA, especially paying attention to toxicity and tumorigenicity, the results suggesting that MenSCs transplantation is safe for rat model of IUA (9). One meta-analysis systematically reviewed also suggested that MenSCs transplantation is safe for IUA treatment by prolonging menstruation duration and recovering endometrial thickness (45). However, research on the safety of MenSCs transplantation is not enough, and whether graft-vs.-host disease, adverse reactions or malignant transformation will occur after transplantation remains to be observed for a long time. More researches on safety of stem cell transplantation are needed to achieve the goals of MenSCs transplantation for clinical treatment.

## Conclusion

MenSCs are easily obtained and can self-renew without forming tumors. Based on the multiple biological characteristics of MenSCs, including antifibrosis, angiogenesis, anti-inflammation and immunoregulation properties, MenSCs transplantation seems to be a promising therapy for some diseases. However, most studies have been conducted on animals, and clinical trials are scarce. There are some difficulties for clinical transplantation. The perfect method of obtaining MenSCs effectively needs deep consideration. In addition to MenSCs, bone marrow mesenchymal stem cells, adipose mesenchymal stem cells, human umbilical cord blood mesenchymal stem cells and other stem cells from different sources also play a key role in endometrial regeneration and reconstruction. The best time, method and dosage of stem cell transplantation also need a large sample of clinical data to further verify the safety and effectiveness of MenSCs. Whether the disease could recur after stem cell transplantation. The molecular mechanisms of the cells' angiogenesis, antifibrosis, anti-inflammation and immunoregulation properties need to be explore deeply. In addition, standard methods of sample collection should be optimized, and age-related effects should be investigated. In summary, further preclinical research is essential to achieve the goal of MenSCs transplantation in the clinic.

## Author Contributions

YHe was responsible for writing the first draft of the manuscript. YHa contributed to the data acquisition of the article and revising it critically for important intellectual content. YY was responsible for critical review of the manuscript. All authors read and approved the final manuscript.

## Conflict of Interest

The authors declare that the research was conducted in the absence of any commercial or financial relationships that could be construed as a potential conflict of interest.

## Publisher's Note

All claims expressed in this article are solely those of the authors and do not necessarily represent those of their affiliated organizations, or those of the publisher, the editors and the reviewers. Any product that may be evaluated in this article, or claim that may be made by its manufacturer, is not guaranteed or endorsed by the publisher.

## Abbreviations

IUA: Intrauterine adhesion
MenSCs: Menstrual blood-derived stem cells
TGF-β: Transforming growth factor-β
PDGF-BB: Platelet-derived growth factor
EGF: Epidermal growth factor
VEGFR: Vascular endothelial growth factor Receptor
MCP1: Monocyte chemoattractant protein 1
GMCSF: Granulocyte macrophage colony stimulating factor
SDF-1: Stromal cell-derived factor-1
IGF-1: Insulin-like growth factor 1
CXCR4: CXC chemokine receptor 4
IL-10: Interleukin−10
NK cells: Nature killer cells
HIF-1α: Hypoxia inducible factor-1 alpha
NO: Nitric oxide
IGF: Insulin-like growth factor.
